# Eccentric overload differences between loads and training variables on flywheel training

**DOI:** 10.5114/biolsport.2023.122483

**Published:** 2023-04-05

**Authors:** Alejandro Muñoz-López, Fábio Yuzo Nakamura, Marco Beato

**Affiliations:** 1 Departamento de Motricidad Humana y Rendimiento Deportivo, University of Seville, Seville, Spain; 2 Research Center in Sports Sciences, Health Sciences and Human Development, CIDESD, University of Maia, Maia, Portugal; 3 School of Health and Sports Science, University of Suffolk, Ipswich, United Kingdom

**Keywords:** Eccentric training, Injury prevention, Resistance training, Strength, Squat

## Abstract

There is considerable debate about the existence of a real eccentric overload in flywheel exercises. This study aimed to analyse the differences in concentric: eccentric mechanical output ratios between different loads and variables in the flywheel squat exercise. Twenty physically active men (22.9 ± 2.2 years, height: 1.8 ± 0.1 m, weight: 79.6 ± 8.2 kg) performed a loading test using five moments of inertia. Angular speed was measured using a rotary encoder, while the vertical force was measured using force plates. For each variable (angular speed, angular acceleration, power, vertical force, and torque), mean and peak values were calculated for concentric and eccentric phases to allow comparisons across the loads. We tested the possible differences in Load × Phase (concentric and eccentric) and Load × Variable. The level of significance was established as p < 0.05. A significant Load × Phase interaction was found in mean angular speed, peak vertical force, peak angular acceleration, peak power and peak torque. Higher eccentric overload values were observed with speed-derived variables (angular speed, angular acceleration and power). In conclusion, speed-derived peak variables and lower loads are more likely to show an eccentric overload and can be used to monitor responses to flywheel training.

## INTRODUCTION

Athletes must move in all directions and with frequent changes of direction to meet the performance needs of their sport [1]. These movements are carried out at different speeds and may require high-intensity accelerations and decelerations, which can impact physical loading and fitness [2]. There are different training equipment to improve acceleration or deceleration abilities; for instance, traditional resistance training can improve acceleration ability (i.e., the initial part of a sprinting action) [3]. In contrast, flywheel resistance training devices have mostly been used in training programs to improve performance during the eccentric phase of the movement [4]. With these devices, the athlete first pulls a rope or a strap at the desired (i.e., maximal) intensity, thus spinning a flywheel disk during the concentric phase. Then the rope or strap recoils around the rotary shaft during the eccentric phase, where the athlete is instructed to decelerate the kinetic energy produced during the concentric phase [5].

During flywheel resistance training, practitioners try to achieve a so-called eccentric overload with their athletes, which can be explained as the production of a greater mechanical output during the eccentric phase compared to the concentric one [6, 7]. In a recent meta-analysis, Muñoz-López et al. [8] showed that most of the research that used flywheel resistance training with the purpose to achieve an EO neither measured it nor achieved that. EO can be easily measured using the eccentric:concentric ratio (E:C) [9]. E:C is a parameter used to quantify the application of kinetic variables (e.g., force) during the eccentric phase of a movement in comparison to the concentric phase [6]. Therefore, if the kinetic energy measured is higher during the eccentric phase than the concentric phase, an E:C would be greater than 1 and EO would be achieved [6]. However, a known problem related to EO during flywheel resistance training is its accurate quantification and its reliability [7, 10]. The absence of an EO during flywheel resistance training can be related to the following reasons: 1) the participant is not able to decelerate the kinetic energy towards the end of the eccentric phase, 2) low experience or poor familiarization of the participants with flywheel resistance training [5], 3) the proper selection of the mechanical output used to monitor it [8], or 4) the external load used [11, 12].

An effective selection of the inertial load may be critical to induce EO [13]. Some authors showed significant differences in speed [14–16], power [11, 15, 17], angular acceleration [14, 17], or vertical force [16, 17] between different moments of inertia in flywheel squat exercises. Some authors reported that the lower the moment of inertia, the higher the speed [11, 14, 16]. For the leg extension exercise, Martinez-Aranda and Fernandez-Gonzalo [12] showed that EO is more likely to be produced with a moment of inertia of 0.0375 kg · m^2^. However, they calculated the EO only using peak force, while Muñoz-López et al. [8] showed an influence of the variable selected when the occurrence of EO is assessed. Sabido et al. [11] showed that a lower moment of inertia (i.e., 0.025 kg · m^2^) resulted in higher concentric peak power outputs, whereas higher inertial loads (i.e., 0.075 kg · m^2^) were more likely to produce higher eccentric peak power outputs. However, other researchers reported that a generalization about the use of moments of inertia for all exercises is not possible because mechanical outputs are very dependent on the exercise selected, the characteristics of the athletes and their previous familiarization with the devices [7]. Therefore, it is impossible to state that lower moments of inertia are more suitable for EO production because several factors can affect kinetic and kinematic parameters in both concentric and eccentric phases [7].

A common approach to understanding the neuromuscular profile for a given exercise is to study the force-velocity profile [18]. Typically, the force-velocity profile is calculated using the force and speed of the concentric phase using several external loads in a progressive loading test. Although it has been widely studied with stack machines and free weights (here categorized as traditional resistance equipment), to our knowledge, only a few studies have analyzed it using flywheel resistance training devices [18, 19], reporting both concentric and eccentric outputs. Flywheel devices are equipped with a rotary encoder instead of a linear encoder (used with traditional resistance equipment), which can help to understand better the mechanical rotation demands of both concentric and eccentric phases. To the best of our knowledge, no study has yet investigated the force-velocity relationships using angular variables (i.e., angular speed or torque), nor have investigated those relationships during the eccentric phase of the exercise.

Considering the increasing interest in implementing flywheel resistance training in strength and conditioning programs, especially due to the opportunities to increase muscle activation [20], acute performance, and chronic adaptations [21], it is important to know how to monitor EO over a range of moments of inertia effectively. Therefore, this study aimed to determine the differences in EO between the moments of inertia and the mechanical outputs used in a flywheel squat, which is one of the preferential exercises implemented in those programs. A secondary objective was to compare the force-velocity profile of the concentric and eccentric phases of this exercise. We hypothesized that, first, the kinetic and kinematic outputs will vary across the loads used, and second, the EO level is variable- and load-dependent.

## MATERIALS AND METHODS

### Experimental Approach to the Problem

This study used a randomized observational design to measure the mechanical output during the concentric and the eccentric phase of the flywheel squat exercise using different loads. Subjects performed a randomized loading test using five different moments of inertia (e.g., *loads*). Before testing, subjects conducted two familiarization sessions across all the loads used during the test.

### Subjects

A total of 25 physically active males participated in this study (age: 22.9 ± 2.2 years, height: 1.8 ± 0.1 m, weight: 79.6 ± 8.2 kg). The main inclusion criteria were having a minimum of two years of experience in the squat exercise and having participated for a minimum of 6 months in weekly resistance training programs involving the lower body muscles. At the beginning of the familiarization sessions, none of the subjects had experience executing flywheel squat exercise. We also instructed the subjects not to change their daily nutritional habits or consume any ergogenic aid at least 48 h before testing execution. The minimum sample size required for the statistical test used (two-way repeated-measures ANOVA), considering 80% statistical power, an alpha error of 0.05, and a moderate f effect size of 0.3, was 16 subjects (Power = 0.84). The study protocol followed the guidelines of the Declaration of Helsinki and was approved by the local ethics committee.

### Intervention

Prior to the execution of the tests, the subjects warmed up for 10 minutes. The warm-up consisted of 5 minutes of cycling on an electronic ergometer at a self-selected submaximal pace, followed by mobilization exercises for the ankle, knee, hip, and trunk joints for 5 minutes. Furthermore, the subjects ended the warm-up with five countermovement jumps and six submaximal repetitions of the harness squat exercise with the lowest load used in the tests.

The squat exercise was performed on a cylindrical shaft flywheel resistance device (kBox 3, Exxentric, AB Bromma, Sweden). The loading test consisted of 5 sets (one set for each load in random order), with 5 minutes of rest interspersing the sets. The loads used for each set were 0.025 kg · m^2^, 0.050 kg · m^2^, 0.075 kg · m^2^, 0.100 kg · m^2^, 0.125 kg · m^2^. The radius width of the cylinder was 0.025 m. Two submaximal repetitions were used to accelerate the flywheel disk initially. From the third repetition (the first for further analyses), subjects had to accelerate the flywheel disk at maximum voluntary effort, pulling until the end of the concentric phase. We instructed the participants to decelerate as hard as possible the spinning flywheel disk at the end during the eccentric phase. Only subjects who committed to completing the five loads were considered (n = 20); therefore, 5 subjects were excluded.

Data were recorded using a rotary encoder (EMS22Q, Bourns, Riverside, CA, USA) connected to the flywheel shaft and a prototype multichannel acquisition system (SmartCoach MultiChannel, SmartCoach Technologies Inc., Pleasanton, CA, USA), with specific computer software (SmartPlot V4.7.0, SmartCoach Technologies Inc., Pleasanton, CA, USA). We calculated the mean and peak values for the speed, acceleration, power and torque variables from the raw speed captured for each concentric and eccentric phase movement. In addition, we recorded the vertical ground reaction force using two force platforms (SmartCoach Europe AB, Stockholm, Sweden), synchronized and connected to the SmartCoach Multichannel. From the force platforms, we calculated the mean and peak force. The sampling rate was set at 100 Hz for both capture systems. Finally, we calculated the E:C value as the eccentric phase value divided by the concentric phase value. The average from the three fastest repetitions of each set (i.e., with the highest concentric speed) was considered for further analysis. Further information on data acquisition and computation is published elsewhere [17].

### Statistics

Data are shown as mean ± standard deviation (SD). Data normality was checked using the Shapiro-Wilk test. The sphericity assumption was tested using Mauchly’s sphericity test and corrected using the Greenhouse-Geisser correction in the case of being positive. We used a two-way ANOVA repeated measures test with two within-subjects factors (Load (i.e., moments of inertia) × Phase (i.e., concentric or eccentric)). In addition, we tested the differences between loads in the E:C using a two-way ANOVA repeated measures test with two within-subjects factors (Load × Variable). When a significant interaction was found in both cases, we performed a post hoc analysis using the Bonferroni correction. Alpha level to determine significant differences was 5%, therefore *p* = 0.05. We used the partial eta squared (η^2^_p_) to measure the effect size of the differences for the interactions such as 0.01–0.059 (small), 0.06–0.139 (medium), and > 0.14 (large). We used the Cohen’s *d* to measure the effect size of the post-hoc differences as follows: < 0.20 (trivial), 0.2–0.59 (small), 0.6–1.19 (moderate), 1.2–2.0 (large), and > 2.0 (very large)[22]. Finally, we calculated and plotted the mean ± SD of force, torque, power, and velocity to represent different mechanical relationships for each movement phase. We used the JASP software for Windows (JASP Team, Version 0.16) for all statistical tests.

## RESULTS

### Concentric and eccentric outputs

The mean and peak values for each variable during the concentric and eccentric phases are shown in Table 1 and Table 2, respectively.

**TABLE 1:** Descriptive mean variables and differences for Load, Phase, and Load × Phase. CON = concentric phase. ECC = eccentric phase.

Variable	Load (kg · m^2^)										Differences		
	0.025		0.050		0.075		0.100		0.125		Load	Phase	Load × Phase
	CON	ECC	CON	ECC	CON	ECC	CON	ECC	CON	ECC			
Angular speed (rad/s)	99.6 ± 14.4	96.7 ± 10.9	75.5 ± 8.0	80.3 ± 9.3	63.5 ± 7.2	67.6 ± 8.1	54.7 ± 5.8	61.1 ± 8.6	48.5 ± 5.4	53.8 ± 7.0	p < 0.001 η^2^_p_ = 0.93	p < 0.001 η^2^_p_ = 0.62	p < 0.001 η^2^_p_ = 0.45
Angular acceleration (rad/s^2^)	189.7 ± 19.0	209.0 ± 44.9	116.9 ± 17.8	129.4 ± 22.1	81.7 ± 14.2	92.9 ± 20.1	61.8 ± 12.9	68.9 ± 19.4	51.8 ± 9.8	57.1 ± 9.8	p < 0.001 η^2^_p_ = 0.55	p < 0.001 η^2^_p_ = 0.57	p = 0.081 η^2^_p_ = 0.13
Power (W)	433.4 ± 86.8	469.3 ± 149.5	416.8 ± 103.7	450.6 ± 117.2	366.5 ± 93.8	407.7 ± 123.1	323.3 ± 93.8	358.1 ± 124.7	299.6 ± 86.5	327.2 ± 118.4	p < 0.001 η^2^_p_ = 0.58	p < 0.001 η^2^_p_ = 0.47	p = 0.936 η^2^_p_ = 0.01
Torque (N · m)	4.7 ± 0.5	5.2 ± 1.1	5.8 ± 0.9	6.5 ± 1.1	6.1 ± 1.1	7.0 ± 1.5	6.2 ± 1.3	6.9 ± 2.0	6.5 ± 1.2	7.1 ± 1.9	p < 0.001 η^2^_p_ = 0.55	p < 0.001 η^2^_p_ = 0.57	p = 0.695 η^2^_p_ = 0.03
Vertical force (N)	1483.5 ± 124.3	1371.6 ± 156.0	1582.6 ± 203.9	1452.8 ± 198.2	1604.7 ± 250.9	1458.7 ± 231.5	1583.5 ± 249.0	1434.2 ± 272.3	1618.6 ± 266.5	1454.2 ± 284.0	p < 0.001 η^2^_p_ = 0.19	p = 0.012 η^2^_p_ = 0.93	p = 0.287 η^2^_p_ = 0.06

**TABLE 2:** Descriptive peak variables and differences for Load, Phase, and Load × Phase. CON = concentric phase. ECC = eccentric phase.

Variable	Load (kg · m^2^)										Differences		
	0.025		0.050		0.075		0.100		0.125		Load	Phase	Load X Phase
	CON	ECC	CON	ECC	CON	ECC	CON	ECC	CON	ECC			
Angular speed (rad/s)	161.3 ± 17.6	158.9 ± 18.3	129–0 ± 12.6	127.4 ± 13.7	110.0 ± 10.3	108.4 ± 11.1	96.5 ± 10.6	95.7 ± 11.5	86.7 ± 9.0	85.5 ± 9.2	p < 0.001 η^2^_p_ = 0.95	p < 0.001 η^2^_p_ = 0.71	p = 0.672 η^2^_p_ = 0.03
Angular acceleration (rad/s^2^)	323.5 ± 51.2	380.2 ± 71.6	182.5 ± 25.4	248.7 ± 51.0	133.7 ± 20.6	172.8 ± 45.4	100.5 ± 14.9	125.3 ± 31.0	84.8 ± 12.6	104.6 ± 25.4	p < 0.001 η^2^_p_ = 0.95	p < 0.001 η^2^_p_ = 0.82	p < 0.001 η^2^_p_ = 0.35
Power (W)	754.6 ± 166.5	921.1 ± 298.1	800.0 ± 184.4	835.0 ± 255.0	735.8 ± 188.9	774.3 ± 231.0	666.6 ± 207.4	696.0 ± 220.7	647.5 ± 194.6	622.2 ± 218.1	p = 0.027 η^2^_p_ = 0.46	p < 0.001 η^2^_p_ = 0.23	p < 0.001 η^2^_p_ = 0.23
Torque (N · m)	8.1 ± 1.3	9.5 ± 1.8	9.1 ± 1.3	12.4 ± 2.6	10.0 ± 1.6	13.0 ± 3.4	10.0 ± 1.5	12.5 ± 3.1	10.6 ± 1.6	13.1 ± 3.0	p < 0.001 η^2^_p_ = 0.57	p < 0.001 η^2^_p_ = 0.79	p = 0.009 η^2^_p_ = 0.16
Vertical force (N)	1671.7 ± 140.5	1613.6 ± 168.3	1797.7 ± 195.5	1691.8 ± 223.1	1911.7 ± 270.8	1735.5 ± 272.6	1958.1 ± 269.6	1688.7 ± 262.1	2039.2 ± 315.2	1731.8 ± 313.1	p < 0.001 η^2^_p_ = 0.41	p < 0.001 η^2^_p_ = 0.81	p < 0.001 η^2^_p_ = 0.44

From the mean variables, only the Mean Angular Speed (*p < 0.001, η*^2^_p_ *= 0.45 – large*) showed a significant Load × Phase interaction. In addition, the eccentric Mean Angular Speed was significantly higher than concentric Mean Angular Speed for 0.050 kg · m^2^ (*p < 0.001, d = 0.54 – moderate*), 0.075 kg · m^2^ (*p < 0.05, d = 0.48 – small*), 0.100 kg · m^2^ (*p < 0.001, d = 0.72 – moderate*), and 0.125 kg · m^2^ (*p < 0.001, d = 0.60 – moderate*).

The Peak Vertical Force (*p < 0.001, η*^2^_p_ *= 0.44 – large*), the Peak Angular Acceleration (*p < 0.001, η*^2^_p_ *= 0.35 – large*), the Peak Power (*p < 0.001, η*^2^_p_ *= 0.23 – large*), and the Peak Torque (*p = 0.009, η*^2^_p_ *= 0.16 – large*) showed a significant Load × Phase interaction. In addition, the Peak Vertical Force concentric outputs were higher than the eccentric outputs at 0.075 kg · m^2^ (*p < 0.001, d = 0.70 – moderate*), 0.100 kg · m^2^ (*p < 0.001, d = 1.08 – large*), and 0.125 kg · m^2^ (*p < 0.001, d = 1.23 – large*). For the Peak Angular Acceleration, eccentric outputs were higher than concentric outputs for 0.025 kg · m^2^ (*p < 0.001, d = 1.44 – large*), 0.050 kg · m^2^ (*p < 0.001, d = 1.68 – large*), 0.075 kg · m^2^ (*p < 0.001, d = 0.99 – large*), and 0.100 kg · m^2^ (*p < 0.05, d = 1.02 – large*). For the Peak Power, the eccentric outputs were higher than concentric outputs for 0.025 kg · m^2^ (*p < 0.001, d = 0.76 – moderate*).

### Eccentric:concentric ratio between variables differences

**FIG. 1:**
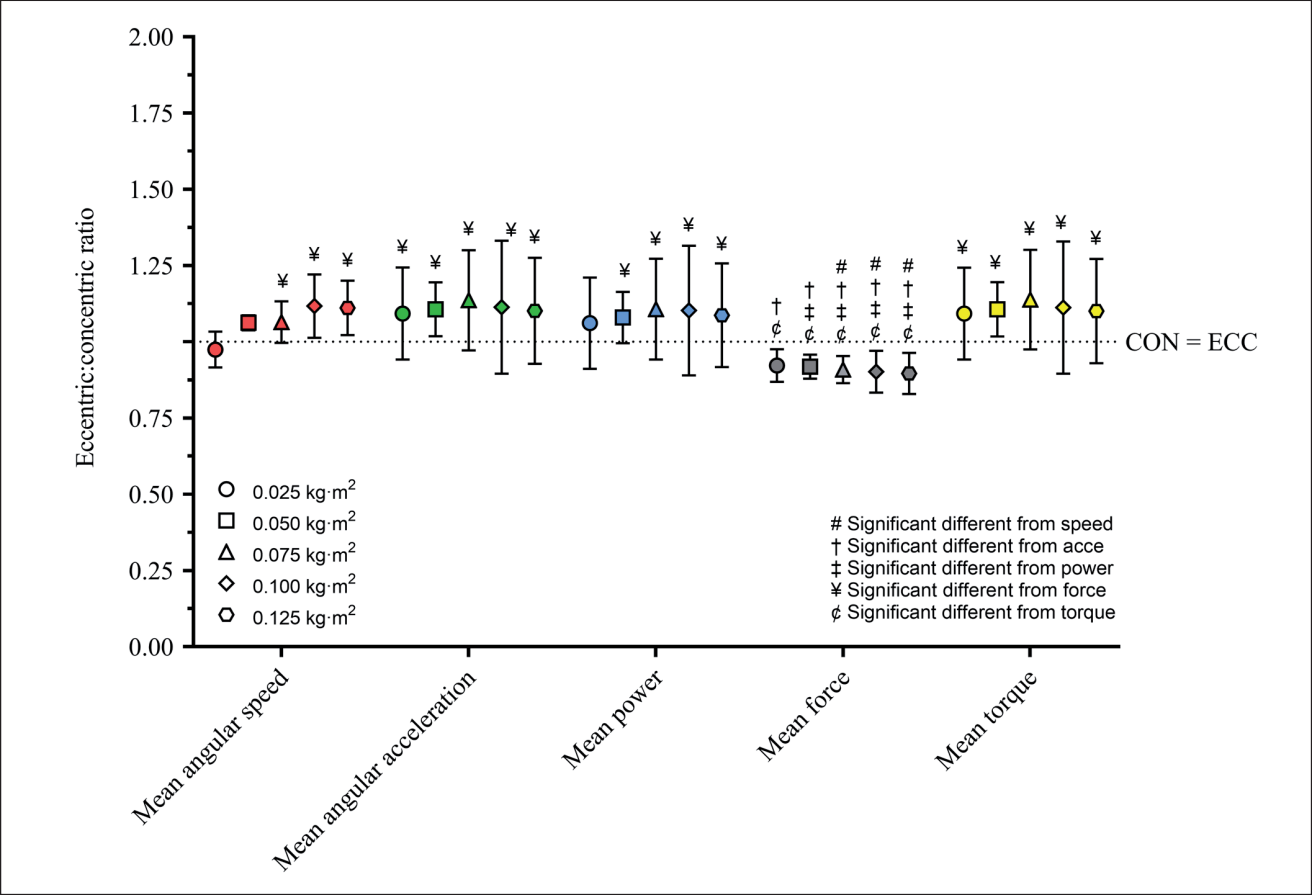
Eccentric:concentric ratio for each moment of inertia and mechanical variable mean values. The dotted horizontal line represents a ratio equal to 1. Values above this line represents an eccentric overload.

**FIG. 2:**
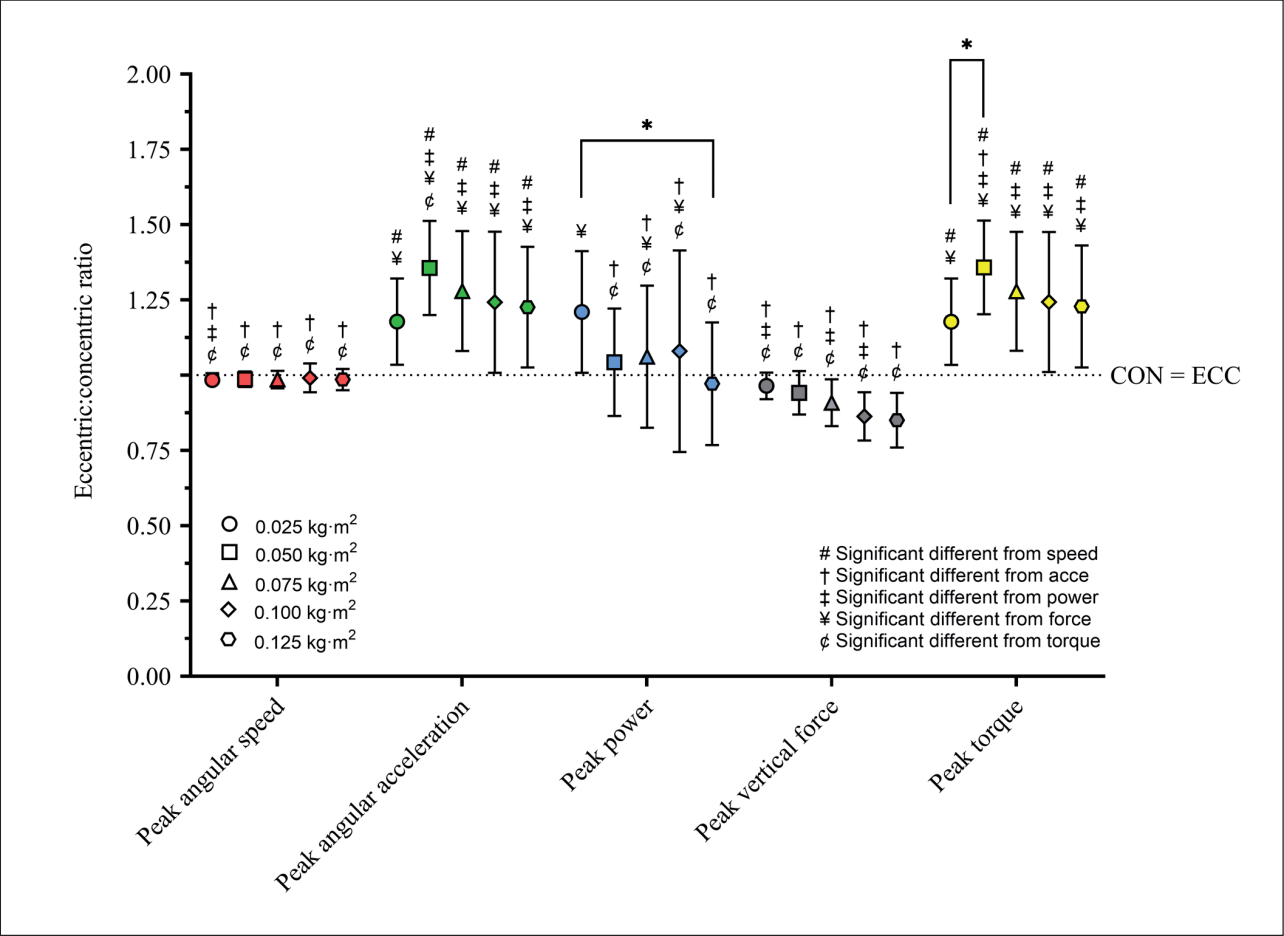
Eccentric:concentric ratio for each moment of inertia and mechanical variable peak values. The dotted horizontal line represents a ratio equal to 1. Values above this line represents an eccentric overload.

Figures 1 and 2 show the E:C for the mean and peak variables, respectively. The mean and peak variables did not show a significant Load effect, but a Variable significant effect (*p < 0.001*) was detected. For mean variables, E:C was higher than 1 (i.e., eccentric overload) for Mean Angular Acceleration, Mean Torque, Mean Power, and Mean Angular Speed, but lower than 1 (i.e., no eccentric overload) for Mean Vertical Force. For peak variables, E:C was higher than 1 for Peak Acceleration, Peak Torque, and Peak Power, but lower than 1 for Peak Angular Speed and Peak Vertical Force. Only the peak variables showed a Load × Variable significant interaction (*p < 0.001, η*^2^_p_ *= 0.26 – large*). In this case, Peak Power showed a significant difference between 0.025 kg · m^2^ and 0.125 kg · m^2^, and Peak Torque between 0.025 kg · m^2^ and 0.050 kg · m^2^.

### Mechanical profiles

**FIG. 3:**
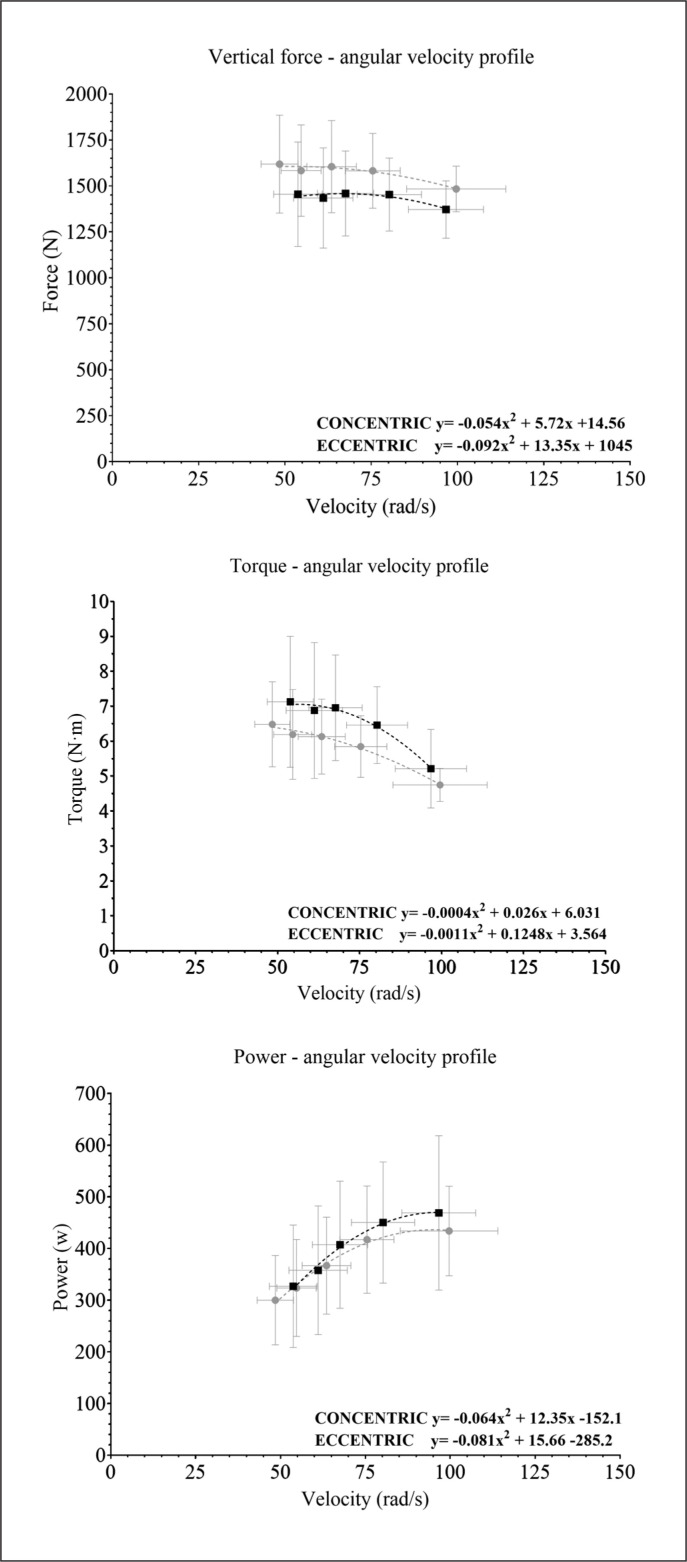
Force-, Torque-, and Power-Velocity profiles for the concentric (black squares) and eccentric (grey circles) phases. The vertical and the horizontal lines represents the individuals standard deviations for each y-variable (vertical lines) and x-variable (horizontal lines).

Figure 3 shows the mechanical profile between the Vertical Force-Angular Velocity, Torque-Angular Velocity, and Power-Angular Velocity for the concentric and eccentric phases. The concentric Vertical Force-Angular Velocity profile was higher than the eccentric profile for all the load ranges studied. The opposite was shown for the Torque-Angular Velocity profile. Finally, the Power-Angular Velocity profile was higher for the concentric phase, but the velocity was higher. Although the Force-Angular Velocity profile was almost linear, the Torque- and Power-Angular Velocity profiles showed a curvilinear relationship.

## DISCUSSION

This study aimed to determine the differences in EO between the moments of inertia and the mechanical outputs used in a flywheel squat. A secondary objective was to compare the force-velocity profile of the concentric and eccentric phases of this exercise. In this research, we showed that 1) EO is recorded preferentially when peak variables are used instead of mean variables; 2) speed derived variables (i.e., angular speed, angular acceleration, power) are more likely to show higher eccentric than concentric outputs, compared to vertical force; 3) the E:C is a suitable parameter to measure EO, with the capability to distinguish levels of EO between loads, especially when peak power is used; 4) the torque-angular velocity profile resulted in a curvilinear relationship, with higher concentric outputs compared to eccentric outputs along the load range studied.

We showed that mean and peak angular acceleration followed the same response pattern across the loads for the first time. As with other training equipment, flywheel resistance devices showed lower peak and mean velocities at higher moments of inertia (4, 14, 23, 35). We found a similar response even though we monitored angular velocity instead of linear velocity as done in other studies (4, 14, 23, 35). In agreement with previous results (13, 25, 35), the mean and peak power decreased when the moment of inertia increased. Furthermore, the angular acceleration showed the largest differences considering Load and Phase. Similar differences were observed with angular acceleration but during leg extension exercise [14]. However, the higher the load, the higher the mean or peak torque measured. Finally, the mean vertical force did not significantly change across loads. Instead, the peak vertical force increased when higher loads were used (Table 2). Similar mean vertical forces were previously observed between different loads in flywheel exercises [16, 18]. For that reason, practitioners may benefit from using low moments of inertia to improve acceleration ability (i.e., 0.025 kg · m^2^), which will maximize both concentric and eccentric power outputs, despite the fact that force or torque outputs were lower in those cases. On the other hand, if the intention is to generate high peak force values in both eccentric and concentric phases, it is advisable to use higher moments of inertia, as reported in this study.

Several studies showed the relevance of using flywheel devices in strength and conditioning programs due to their capacity to enhance the eccentric load and related adaptations [5, 23, 24]. Maroto et al. [23] showed the largest changes for Peak Power during the eccentric phase, compared to the concentric phase, after 12 weeks of training using a single-leg flywheel squat exercise. This resulted in moderate increases in tight muscle volume and large increases in maximum repetition and vertical jump performance. Furthermore, increased muscle volume (up to 10%) was observed in the quadriceps muscles during leg extension exercise using a cylindrical flywheel device after 5 weeks of training [24]. Tous-Fajardo et al. [5] introduced the concept of E:C for measuring EO, and subsequently Sabido et al. [11] showed that when E:C was calculated using peak power, the highest values were observed with moments of inertia close to 0.075 kg · m^2^, using a cylindrical flywheel quarter squat exercise. In the current study, we show that whether EO is achieved or not depends on the variable and load selected (Figures 1 and 2). Mean variables showed E:C higher than 1, despite only mean angular speed showed a significant difference CON and ECC, probably explained by the high individual variance in these variables. Furthermore, we showed that the peak values showed greater differences between the loads than the mean values and the highest E:C was observed when peak angular acceleration was used to calculate it. However, Peak Power was also sensible to detect differences between the lower and the highest load used to monitor the EO (Figure 2). Despite peak variables were more sensible to show EO, it is possible that at an individual level, mean variables could also show that. It can be explained by a lower sensibility of the peak variables to show differences in the execution technique, and as a result a higher advantage for monitoring EO using peak variables. More research is needed to confirm these results.

When E:C was calculated with Peak Vertical Force, it decreased when loads increased but it was never higher than 1, which means that no EO was achieved using this variable. To achieve high levels of EO, low inertial loads (i.e., 0.025 kg · m^2^) may also be used. However, in this research EO could also be observed up to 0.100 kg · m^2^. Hence Peak Power EO can be achieved across a broad range of loads and the choice of the load depends on the aims of the strength and conditioning program (e.g., focus on speed or force production). Curiously, Sabido et al. [11] found the Peak Power EO close to 0.075 kg · m^2^, and it might be related to the fact that they used quarter squat while we used half squat. The effect of how deep participants squat in the flywheel exercise on the occurrence of EO needs to be clarified in the future.

To monitor the neuromuscular profile of an athlete, for instance, the force-velocity profile can be used, which shows the relationship between both mechanical variables, reflecting how much force an athlete could exert for a given velocity [25–27]. Profiling exercises can help develop individualized training programs using the slope of the relationship [15] and monitor changes in performance [27]. Typically, these profiles are measured using traditional resistance exercises, where a linear relationship is shown between force and velocity [25]. However, the relationship between power and velocity is curvilinear [25]. In addition, those profiles are calculated during the concentric phase of the movement only. Our results suggest a similar profile for the flywheel squat exercise for concentric and eccentric outputs. This is explained by the very low differences observed between the vertical force loads, which agrees with previous results [16, 18]. On the contrary, greater slopes were observed when linear velocity was used instead of angular velocity in flywheel devices [19]. Although measuring flywheel device performance using the mechanics of linear momentum may help calculate a force-velocity profile slope, it is mostly unpractical and difficult to set a linear encoder, instead, a rotary encoder could be used to measure torque instead of force [7]. In fact, torque can be understood as the equivalent of force exerted on the angular momentum. Furthermore, the force cannot be easily calculated from the speed during flywheel exercises because the width of the shaft varies along with the range of motion of exercise [28]. Our results showed a curvilinear relationship between torque and angular velocity, with higher concentric outputs for all the loads measured. Finally, the power-angular velocity relationship showed that the largest power output was observed at higher velocities (Figure 3). Indeed, those values were higher for the concentric phase, but the lower the angular speed, the smallest the difference between both phases. Monitoring the power-angular velocity may help decide which load may be used for training programs when using flywheel devices to enhance acceleration and deceleration abilities, which is still an underexplored topic [29].

This study has some limitations. First, our results may only apply to squat exercises. Second, the type of flywheel shaft influences the mechanical output, regardless of the moment of inertia [30]. Lastly, this study enrolled a sample of active males participated. Therefore, these findings need to be confirmed in other populations (i.e., female) and different sport levels (e.g., professional athletes). Future research is needed to verify the reliability of these parameters in sports, to verify our findings using different exercises such as flywheel leg curl, flywheel leg press, etc, and also with other flywheel devices (i.e., conical shaft shape).

## CONCLUSIONS

In conclusion we found that EO depends on the mechanical output and discrete variables analyzed (peak variables and speed derived variables showed higher EO). Lastly, the torque-angular velocity profile resulted in a curvilinear relationship, with higher concentric outputs than eccentric outputs along with the studied load range. The E:C is a suitable parameter to measure EO, with the capability to distinguish levels of EO between loads, especially when peak power parameters are used.

## References

[1] de Hoyo M, Sañudo B, Carrasco L, et al. Effects of 10-week eccentric overload training on kinetic parameters during change of direction in football players. J Sports Sci. 2016; 34(14):1380–1387. 26963941 10.1080/02640414.2016.1157624

[2] Beato M, Drust B. Acceleration intensity is an important contributor to the external and internal training load demands of repeated sprint exercises in soccer players. Res Sports Med. 2021; 29(1):67–76. 32200649 10.1080/15438627.2020.1743993

[3] Rumpf MC, Lockie RG, Cronin JB, et al. Effect of Different Sprint Training Methods on Sprint Performance Over Various Distances: A Brief Review. J Strength Cond Res. 2016; 30(6):1767–1785. 26492101 10.1519/JSC.0000000000001245

[4] Suchomel TJ, Wagle JP, Douglas J, et al. Implementing Eccentric Resistance Training—Part 1: A Brief Review of Existing Methods. J Funct Morphol Kinesiol. 2019; 4(2):38. 33467353 10.3390/jfmk4020038 PMC7739257

[5] Tous-Fajardo J, Maldonado RA, Quintana JM, et al. The flywheel leg-curl machine: offering eccentric overload for hamstring development. Int J Sports Physiol Perform. 2006; 1(3):293–298. 19116442 10.1123/ijspp.1.3.293

[6] Berg HE, Tesch PA. Force and power characteristics of a resistive exercise device for use in space. Acta Astronaut. 1998; 42(1):219–230. 11541605 10.1016/s0094-5765(98)00119-2

[7] Maroto-Izquierdo S, Raya-González J, Hernández-Davó JL, et al. Load Quantification and Testing Using Flywheel Devices in Sports. Front Physiol. 2021; 12(October):1–5. 10.3389/fphys.2021.739399 PMC8587883 34777007

[8] Muñoz-López A, Fonseca F, Ramirez-Campillo R, et al. The use of real-time monitoring during flywheel resistance training programs: how can we measure the eccentric overload? A systematic review and meta-analysis. Biol Sport. 2021;639–652. 10.5114/biolsport.2021.101602 PMC8670814 34937974

[9] Nuñez FJ, Hoyo M de, López AM, et al. Eccentric-concentric Ratio: A Key Factor for Defining Strength Training in Soccer. Int J Sports Med. 2019; 40(12):796–802. 31434138 10.1055/a-0977-5478

[10] Piqueras-Sanchiz F, Sabido R, Raya-González J, et al. Effects of Different Inertial Load Settings on Power Output Using a Flywheel Leg Curl Exercise and its Inter-Session Reliability. J Hum Kinet. 2020; 74(1):215–226. 33312289 10.2478/hukin-2020-0029 PMC7706644

[11] Sabido R, Hernández-Davó JL, Pereyra-Gerber GT. Influence of different inertial loads on basic training variables during the flywheel squat exercise. Int J Sports Physiol Perform. 2018; 13(4):482–489. 28872379 10.1123/ijspp.2017-0282

[12] Martinez-Aranda LMM, Fernandez-Gonzalo R. Effects of inertial setting on power, force, work, and eccentric overload during flywheel resistance exercise in women and men. J Strength Cond Res. 2017; 31(6):1653–1661. 28538317 10.1519/JSC.0000000000001635

[13] Beato M, dello Iacono A. Implementing Flywheel (Isoinertial) Exercise in Strength Training: Current Evidence, Practical Recommendations, and Future Directions. Front Physiol. 2020; 11. 32581845 10.3389/fphys.2020.00569 PMC7283738

[14] Muñoz-López A, Pozzo M, Floria P. Real-time mechanical responses to overload and fatigue using a flywheel training device. J Biomech. 2021; 121:110429. 33873106 10.1016/j.jbiomech.2021.110429

[15] McErlain-Naylor SA, Beato M. Concentric and eccentric inertia–velocity and inertia–power relationships in the flywheel squat. J Sports Sci. 2021; 39(10):1136–1143. 33337956 10.1080/02640414.2020.1860472

[16] Núñez FJ, Galiano C, Muñoz-López A, et al. Is possible an eccentric overload in a rotary inertia device? Comparison of force profile in a cylinder-shaped and a cone-shaped axis devices. J Sports Sci. 2020; 38(14):1624–1628. 32299296 10.1080/02640414.2020.1754111

[17] Muñoz-López A, Floria P, Sañudo B, et al. The Maximum Flywheel Load: A Novel Index to Monitor Loading Intensity of Flywheel Devices. Sensors. 2021; 1–16. 34884128 10.3390/s21238124 PMC8662394

[18] Spudić D, Smajla D, Šarabon N. Validity and reliability of force–velocity outcome parameters in flywheel squats. J Biomech. 2020; 107:109824. 32517866 10.1016/j.jbiomech.2020.109824

[19] Spudić D, Cvitkovič R, Šarabon N. Assessment and Evaluation of Force – Velocity Variables in Flywheel Squats: Validity and Reliability of Force Plates, A Linear Encoder Sensor, and A Rotary Encoder Sensor. Appl Sci. 2021; 11(22):10541.

[20] Norrbrand L, Pozzo M, Tesch PA. Flywheel resistance training calls for greater eccentric muscle activation than weight training. Eur J Appl Physiol. 2010; 110(5):997–1005. 20676897 10.1007/s00421-010-1575-7

[21] Tesch PA, Fernandez-Gonzalo R, Lundberg TR. Clinical applications of iso-inertial, eccentric-overload (YoYo^TM^) resistance exercise. Front Physiol. 2017; 8:241. 28496410 10.3389/fphys.2017.00241 PMC5406462

[22] Hopkins WG, Marshall SW, Batterham AM, et al. Progressive statistics for studies in sports medicine and exercise science. Med Sci Sports Exerc. 2009; 41(1):3–13. 19092709 10.1249/MSS.0b013e31818cb278

[23] Maroto-Izquierdo S, Fernandez-Gonzalo R, Magdi HR, et al. Comparison of the musculoskeletal effects of different iso-inertial resistance training modalities: Flywheel vs. electric-motor. Eur J Sport Sci. 2019; 19(9):1184–1194. 30957699 10.1080/17461391.2019.1588920

[24] Norrbrand L, Fluckey JD, Pozzo M, et al. Resistance training using eccentric overload induces early adaptations in skeletal muscle size. Eur J Appl Physiol. 2008; 102(3):271–281. 17926060 10.1007/s00421-007-0583-8

[25] Cross MR, Brughelli M, Samozino P, et al. Methods of Power-Force-Velocity Profiling During Sprint Running: A Narrative Review. Sports Medicine. Springer International Publishing; 2017. p. 1255–1269. 10.1007/s40279-016-0653-3 27896682

[26] Morin J-BB, Samozino P. Interpreting Power-Force-Velocity Profiles for Individualized and Specific Training. Int J Sports Physiol Perform. 2016; 11(2):267. 26694658 10.1123/ijspp.2015-0638

[27] Iglesias-Soler E, Fernández-Del-Olmo M, Mayo X, et al. Changes in the force-velocity mechanical profile after short resistance training programs differing in set configurations. J Appl Biomech. 2017; 33(2):144–152. 27918682 10.1123/jab.2016-0181

[28] Sabido R, Hernández-Davó JL, García-Valverde A, et al. Influence of the Strap Rewind Height during a Conical Pulley Exercise. J Hum Kinet. 2020; 74(1):109–118. 33312280 10.2478/hukin-2020-0018 PMC7706643

[29] Beato M, Maroto-Izquierdo S, Hernández-Davó JL, et al. Flywheel Training Periodization in Team Sports. Front Physiol. 2021; 12:1–6. 10.3389/fphys.2021.732802 PMC8606557 34819871

[30] Muñoz-López A, Galiano C, Nuñez FJ, et al. The flywheel device shaft shape determines force and velocity profiles in the half squat exercise. J Hum Kinet. 2022; 81:15–25. 35291636 10.2478/hukin-2022-0002 PMC8884883

